# DEVELOPMENT AND VALIDATION OF A U.S. AND GERMAN SHORT VERSION OF THE LATER LIFE WORKPLACE INDEX (LLWI-S)

**DOI:** 10.1093/geroni/igad104.2391

**Published:** 2023-12-21

**Authors:** Juergen Deller, Julia Finsel, Anne Wöhrmann, Eduardo Oliveira, Hila Axelrad, Michela Vignoli, Xiuzhu Gu, Beatrice Van der Heijden

**Affiliations:** Leuphana University of Lueneburg, Lueneburg, Niedersachsen, Germany; Leuphana University Lüneburg, Lüneburg, Niedersachsen, Germany; Federal Institute for Occupational Safety and Health (BAuA), Dortmund, Nordrhein-Westfalen, Germany; University of Porto, Porto, Porto, Portugal; Interdisciplinary Center Herzliya – IDC, Herzliya, Tel Aviv, Israel; University of Trento, Trento, Trentino-Alto Adige, Italy; Tokyo Institute of Technology, Tokyo, Tokyo, Japan; Radboud University Nijmegen, Nijmegen, Gelderland, Netherlands

## Abstract

Employment of older individuals becomes more important in demographic change. To identify organizational practices that foster the motivation, health, and performance of older employees in particular, an efficient holistic assessment of relevant organizational factors is needed. The 80-item Later Life Workplace Index (LLWI) provides such a measure for organizational practices for older employees by differentiating nine domains. The LLWI has been applied in emerging research in various research contexts and countries. However, it is quite a lengthy measure. Therefore, this paper describes the development and validation of the LLWI-S, a 29-item short measure covering all nine domains in order to achieve a more efficient instrument. The development of the short version was both, judgemental- and statistics-driven. A focus group consisting of the original LLWI-authors and several international scholars, whose research expertise lies in the field of employment and older employees, has developed the LLWI-S. A German- and an English-language version of the LLWI-S have been validated in Germany and the U.S. Reliability and model fit for the U.S. and the German version as well as multi-group CFA for measurement invariance will be presented. Results show that the measures have good internal reliability and model fit. We provide researchers and practitioners with a validated short measure to efficiently assess all nine relevant organizational domains for older employees. Researchers can utilize the LLWI-S to gain a comprehensive understanding of all relevant areas of organizational influences on later life work, while practitioners are able to assess their organizational readiness for an aging workforce.

